# Poly(3-hydroxybutyrate) Modified with Thermoplastic Polyurethane and Microfibrillated Cellulose: Hydrolytic Degradation and Thermal and Mechanical Properties

**DOI:** 10.3390/polym16243606

**Published:** 2024-12-23

**Authors:** Adriana Nicoleta Frone, Denis Mihaela Panaitescu, Augusta Raluca Gabor, Cristian-Andi Nicolae, Marius Ghiurea, Corina Bradu

**Affiliations:** 1 National Institute for Research & Development in Chemistry and Petrochemistry—ICECHIM, 202 Splaiul Independentei, 060021 Bucharest, Romania; adriana.frone@icechim.ro (A.N.F.); raluca.gabor@icechim.ro (A.R.G.); cristian.nicolae@icechim.ro (C.-A.N.); ghiurea@gmail.com (M.G.); 2 Faculty of Biology, University of Bucharest, 91–95 Splaiul Independentei, 050095 Bucharest, Romania; corina.bradu@g.unibuc.ro

**Keywords:** polyhydroxyalkanoates, nanocellulose, elastomeric polyurethanes, hydrolytic degradation, thermogravimetric analysis, scanning electron microscopy

## Abstract

Blending poly(3-hydroxybutyrate) (PHB) with other polymers could be a rapid and accessible solution to overcome some of its drawbacks. In this work, PHB was modified with microfibrillated cellulose (MC) and a thermoplastic polyurethane containing biodegradable segments (PU) by two routes, using a masterbatch and by direct mixing. The PU and MC modifiers improved the thermal stability of PHB by up to 13 °C and slightly decreased its melt viscosity and crystallinity, thus improving the melt processability. The addition of PU in PHB composites led to a decrease in the storage modulus, which did not exceed 20% at room temperature. The hydrolytic degradation in an alkaline environment at 50 °C for 28 days decreased the thermal stability of the composites by 58–65 °C, while the lower mass loss and morphological features showed that the PU modifier delayed the degradation of the PHB composites. The improved thermal stability, melt processability, and lower cost, along with higher flexibility and the possibility of controlling the hydrolytic degradation by the PU content, make the PHB/PU/MC composites obtained by the masterbatch method promising materials for medical and engineering applications.

## 1. Introduction

Poly(3-hydroxybutyrate) (PHB) is an aliphatic polyester produced industrially by microbial biosynthesis. Among polyhydroxyalkanoates (PHA), PHB is the main candidate for substituting synthetic polymers in packaging and biomedical applications [1,2]. PHB is biodegradable and biocompatible and its behavior during melt processing is similar to that of common thermoplastic polymers. However, it is a very brittle material and its price is two to five times higher than that of conventional synthetic polymers [1,2]. Moreover, its brittleness increases during storage due to secondary crystallization [3]. Extensive research has focused on the improvement of PHB’s properties, together with decreasing its price.

Blending PHB with other polymers could provide a rapid and accessible solution to this problem [2]. PHB blends with poly(lactic acid) (PLA), poly(ε-caprolactone) (PCL), medium-chain-length polyhydroxyalkanoates, and other polyesters have been extensively studied and progress has been made in improving some of PHB’s properties [2,4,5,6]. The use of an elastomer as the second polymer in PHB blends may provide important benefits for mitigating both brittleness and high costs. However, blending PHB with natural rubber (NR) and epoxidized NR did not lead to any improvement in properties unless a compatibilizer was used [7]. Elastomeric polyurethanes have also been tested to improve the ductility of PHB [8,9,10,11,12]. Polyurethane (PU) is a versatile polymer that covers a wide range of structures and properties [13]. Although polyurethanes can be degraded by microorganisms under environmental conditions, their degradation is very slow and the degradation products could be toxic [14]. According to the European norm EN 13432 [15] related to biodegradable and compostable materials, PUs are non-biodegradable polymers because they are not able to degrade 90% after 6 months under composting conditions [16]. In general, the degradation rate of segmented PUs is influenced by the chemistry of hard and soft domains, their chain length, the type and number of labile groups, and the degree of crystallinity.

In one of the first attempts to obtain PHB/PU blends, PHB was modified with a cycloaliphatic polyurethane to obtain microporous membranes as pericardial patches [17]. The blends were obtained by casting a mixture of a PU solution in tetrahydrofuran with a PHB solution in chloroform. The obtained membrane stimulated the growth of epithelium according to the histological evaluation of the patches after their implantation and extirpation, but the membrane was stiffer than pericardium [17]. In another study, poly(3-hydroxybutyrate-co-3-hydroxyvalerate) (PHBV) blends with up to 40 wt% PU, showing an increased ductility, were prepared by casting mixed solutions of PHBV in chloroform and PU in dimethylformamide [8]. PHBV/PU blends with up to 50 wt% PU were also prepared by melt mixing and compression molding [9]. These blends were immiscible and showed a continuous decrease in tensile strength and modulus, with an increase in PU content along with an increase in the elongation at break for materials with more than 20 wt% PU in the blend. In addition, disintegration under composting conditions was not affected by the presence of PU at up to 40 wt% in the blend, highlighting the possible application of PHBV/PU blends in packaging [9]. In another attempt, PHBV/PU (100:10 and 100:30) composites containing cellulose (10 or 30 phr) were obtained by twin screw extrusion and injection molding [12]. The obtained PHBV/PU blends showed a matrix-droplet morphology, while the addition of cellulose determined an important decrease in the droplets’ size [12]. The addition of 30 wt% PU increased the toughness of PHBV while the further addition of cellulose partially reduced this effect. Moreover, the composites containing 30 phr cellulose showed an unexpected slowdown of the degradation rate under composting conditions [12].

These adverse effects of PU and cellulose, which have been reported for certain PHBV composites, are intriguing and deserve more rigorous evaluation. Moreover, the use of microfibrillated cellulose (MC) in the PHB/PU blends instead of common cellulose with micrometric widths could bring new benefits due to a similar reinforcing effect at much lower MC concentrations [18]. Microfibrillated cellulose is obtained from cellulose by applying eco-friendly mechanical treatment, without using acid hydrolysis or other chemical processes harmful to the environment, and is characterized by a high aspect ratio and flexibility [18,19]. Therefore, in this work, PHB was modified with MC (2 wt%) and different amounts of a PU containing biodegradable segments. This PU modifier, containing poly(ε-caprolactone) as a soft segment and a low proportion of hard segments, was selected based on previous results showing its good toughening effect in PHBV/PU blends [10]. The influence of MC and PU modifiers on PHB’s thermal and mechanical properties was deeply studied in the present work. The hydrolytic degradation of the PHB/PU/MC composites was also investigated because the addition of the two modifiers could influence PHB’s degradability, as noted in a previous report [12]. The degradation test was conducted in an alkaline environment to ensure accelerated conditions, knowing that the degradation rate of PHB is increased in the presence of hydroxide ions in the aqueous medium [20]. The results obtained following the characterization of PHB/PU/MC composites represent a step forward in the application of these composites in the medical and packaging fields.

## 2. Materials and Methods

### 2.1. Materials

Pelletized PHB was purchased from Biomer (Schwalbach am Taunus, Germany). According to the producer’s data sheet, PHB has a density of 1.20 g/cm^3^, a hardness Shore D of 69, and a Charpy impact strength (unnotched) of 5 J/cm^2^ (ISO 179). Microfibrillated cellulose (MC) was obtained from microcrystalline cellulose (MCC, 20 µm, Sigma Aldrich, Saint Louis, MO, USA) by applying a high-pressure mechanical treatment. Briefly, a 1 wt% MCC suspension in water was mechanically treated for 12 cycles using a microfluidizer processor LM20 (Microfluidics, Westwood, MA, USA). The cellulose gel was frozen at −20 °C for 48 h and freeze-dried (FreeZone 2.5 L, Labconco, Kansas City, MO, USA) at −85 °C and 0.006 mbar for 48 h, producing MC as a powder. A PU containing biodegradable segments and characterized by a molecular weight (M_n_) of 32 kg/mol was obtained from Petru Poni Institute of Macromolecular Chemistry (Iasi, Romania) according to a method described in [10]. Sodium hydroxide was purchased from Merck (Kenilworth, NJ, USA) and used as received.

### 2.2. Preparation of the Composites

A masterbatch containing 28.6 wt% MC was obtained by melt mixing PU with MC in a weight ratio of 5/2 in a 50 cm^3^ Brabender mixing chamber at 65 °C for 8 min using a rotor speed of 60 min^−1^. PHB/PU/MC composites with a PU concentration varying from 5 to 15 wt% were obtained in the Brabender mixing chamber at 165 °C for 7 min at a rotor speed of 40 min^−1^ by adding the masterbatch into the melted PHB and supplementing with PU in the case of the composites with 10 and 15 wt% PU. The same masterbatch amount was added into all the composites for a final MC content of 2 wt% (method I). The names of the composites and final concentrations of MC and PU in the composites are summarized in Table 1. Further, the compounds were shaped into sheets by passing on a laboratory two-roll mill (Polymix 110 L, Duisburg, Germany). The PHB/PU10/MCII composite (without masterbatch) was obtained using the same equipment and conditions by directly mixing PHB with PU and MC (method II, Table 1).

The films used for characterization were obtained by compression molding on an electrically heated press P200E (Dr. Collin, Ebersberg, Germany) at 175 °C, under the following conditions: pre-heating for 120 s at 0.5 MPa, compression for 60 s at 10 MPa, and cooling for 60 s in a cooling cassette without pressure. Neat PHB was also processed in the same conditions and served as a reference.

### 2.3. Characterization

#### 2.3.1. Thermogravimetric Analysis (TGA)

Thermal stability studies were conducted on the prepared composites before and after being subjected to hydrolytic degradation using a TA-Q5000 analyzer (TA Instruments, New Castle, DE, USA) at a heating rate of 10 °C min^−1^ from RT to 700 °C, under a nitrogen flow of 40 mL min^−1^. The maximum degradation temperature (*T_max_*) was taken as the peak temperature from the first derivative of the TGA curves (DTG) while the temperature at 10% weight loss (*T_10%_*) and the residue at 700 °C (*R_700_*) were determined using TA Instrument software, version 5.2.0.

#### 2.3.2. Differential Scanning Calorimetry (DSC)

The thermal properties of the PHB/PU/MC composites were analyzed with DSC Q2000 equipment (TA Instruments, New Castle, DE, USA) under a helium atmosphere. Indium was used for the calibration of temperature and heat capacity. Samples of 11–12 mg were heated from −55 °C to 215 °C, cooled down to −55 °C, and heated again to 215 °C with a heating or cooling rate of 10 °C min^−1^. The samples were maintained for 2 min at the end of the cycles, at −55 °C and 215 °C, respectively, to remove previous thermal history and to ensure temperature equilibration. Modulated DSC was used to determine the glass transition temperature (*T_g_*) of PHB in the following conditions: a modulation period of 40 s and a temperature amplitude of ±1 °C. The degree of crystallinity (*X_c_*) was calculated with the equation:

(1) $$X_{c}=\frac{∆H}{{∆H}_{0}\times w_{PHB}}\times 100$$

where Δ*H* is the melting enthalpy calculated from the DSC curves and Δ*H*_0_ is the theoretical melting enthalpy of 100% crystalline PHB (146 J/g [21]), while *w_PHB_* is the weight fraction of PHB in composites.

#### 2.3.3. Dynamic Mechanical Analysis (DMA)

DMA was carried out using a Q800 DMA analyzer (TA Instruments, New Castle, DE, USA) in multi-frequency strain mode. Specimens of 12.7 mm × 6.9 mm × 0.5 mm were measured from −55 °C to 150 °C using a film tension clamp at a frequency of 1 Hz with a heating rate of 3 °C/min, an oscillation amplitude of 15 μm, and static force of 0.01 N. The changes in the storage modulus (*E′*) and loss factor (tan δ) depending on the temperature were analyzed in particular. The glass transition temperature (*T_g_*) was taken as the temperature corresponding to the tan δ peak.

#### 2.3.4. Scanning Electron Microscopy (SEM) Analysis

SEM analysis was used to study the morphological effects of PU and MC in PHB/PU/MC composites before and after hydrolytic degradation. The SEM images were acquired using a tabletop Hitachi TM4000 plus II scanning electron microscope (Hitachi, Tokyo, Japan) working at an accelerating voltage of 10 kV. For this purpose, the initial PHB and composite films were first cryo-fractured in liquid nitrogen, while the films exposed to hydrolytic degradation were kept as such due to their high brittleness, and then were sputter-coated on their sections or surfaces with a thin layer (5 nm) of gold.

#### 2.3.5. X-Ray Diffraction (XRD)

The crystalline structure of PHB in composites was analyzed by X-ray diffraction at room temperature using a SmartLab diffractometer (Rigaku Corporation, Tokyo, Japan) with a Cu X-ray rotating anode (λ = 0.1541 nm) over a 2θ range from 5° to 40° in the following conditions: an accelerating voltage of 45 kV and an emission current of 200 mA.

#### 2.3.6. Hydrolytic Degradation Tests

The hydrolytic degradation tests of pure PHB and composite films were carried out in 0.1 M sodium hydroxide solution (NaOH, pH = 13) and 0.1 M hydrochloric acid solution (HCl, pH = 1) at 25 °C and 50 °C over a period of 28 days. The films with a thickness of about 0.5 mm and a weight of 10 ± 1 mg were immersed in sealed glass tubes with 10 mL of the testing medium and kept at a constant temperature (Velp FOC 225E thermostat) in the absence of light radiation. The tests were performed in duplicate. At different time intervals, the films were removed from the controlled environment, tamped to eliminate excess liquid, and weighed to determine the weight loss according to Equation (2) [22]:

(2) $$∆m\left(\%\right)=\frac{{m_{t}-m}_{0}}{m_{0}}\times 100$$

where *m*_0_ is the initial mass before immersion and *m_t_* is the mass of the sample after testing at time *t*.

#### 2.3.7. Fourier-Transform Infrared Spectroscopy (FTIR)

FTIR analysis was carried out between 550 and 4000 cm^−1^ on the samples subjected to hydrolytic degradation for 28 days using a Varian 3100 Excalibur spectrophotometer equipped with a PikeMIRacle (ZnSe crystal) accessory for attenuated total reflection (ATR). All measurements were performed at room temperature with 128 scans, with a frequency of 5 kHz and at a resolution of 4 cm^−1^.

## 3. Results and Discussion

### 3.1. Melt Rheology

**Figure 1:**
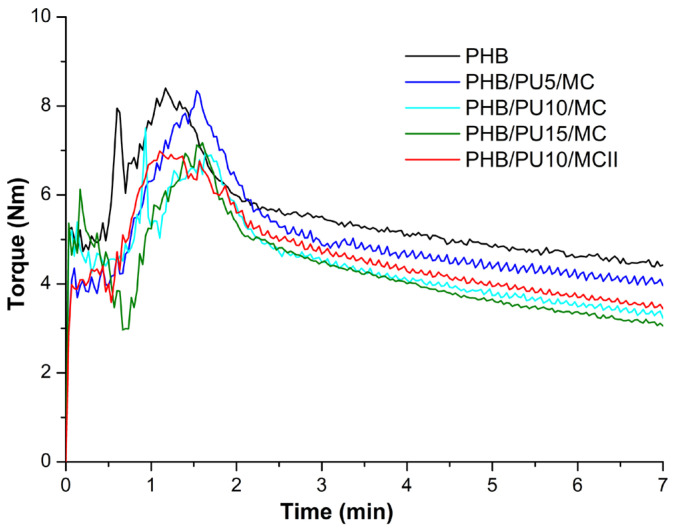
Torque vs. time curves for unmodified PHB and PHB/PU/MC composites.

The melt viscosity of neat PHB and composites can be evaluated using the torque values of the molted materials. Unlike other techniques, torque rheometry is able to estimate and compare the melt viscosity of very different materials, which are characterized by large differences in viscosity, such as neat polymers, their blends, and composites [23]. During processing, the Brabender rheometer records both the torque and the temperature in the material through a sensor located in the mixing chamber. The influence of the two modifiers, PU and MC, on the rheological behavior of the PHB melt can be observed in Figure 1, which shows the torque variation with time during the melt processing of the PHB and composites. The addition of 5 wt% PU in the presence of MC decreased the torque value of PHB composites by 10%, while the use of double the amount of the PU modifier decreased the torque value by 25%. The PU used in these tests has a low molecular weight, which together with its high chain mobility due to the elastomeric nature of polyurethane, has the effect of lowering the composite’s melt viscosity. A decrease in the melt viscosity of PHB was also reported for PHB composites containing nanocellulose and poly(3-hydroxynonanoate) as an elastomeric modifier [24], as well as for PLA blends containing a commercial poly(butylene adipate-co-terephthalate) copolymer in different proportions [25]. Moreover, the decrease in melt viscosity is a common behavior for PHB after the addition of plasticizers, which is a preferred route to improve the melt processability and reduce the thermal degradation of PHB [26]. Therefore, a better thermal stability is expected for the PHB/PU/MC composites as compared to PHB.

### 3.2. Thermal Stability of Composites

**Figure 2:**
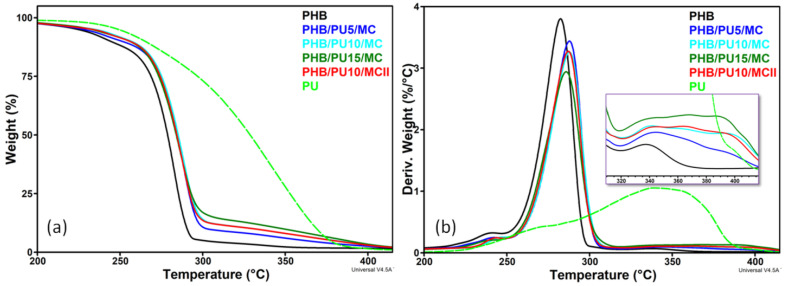
TGA (**a**) and DTG (**b**) curves of PU, PHB, and composites (detail showing the PU degradation in composites).

As seen in Figure 2a, the thermal degradation of the commercial PHB in an inert atmosphere is a multistep process, with an initial weight loss of about 10% between room temperature and 245 °C, which is probably due to the volatilization of a plasticizer, a main degradation step between 250 °C and 300 °C, when there is rapid weight loss due to the accelerated degradation of PHB, followed by a small weight loss of about 4% between 300 °C and 700 °C. The thermal degradation of the PU is a much slower process, which, although it starts at around 200 °C as for PHB, reaches the maximum degradation rate at a temperature that is 60 °C higher than for PHB.

The addition of PU and MC in the PHB matrix improved its thermal stability, shifting the onset of degradation towards higher temperatures (Figure 2a,b). Thus, the temperature at 10% weight loss (*T_10%_*) increased by 7 °C for the PHB/PU5/MC composite compared to the unmodified PHB, and by 11.2–12.5 °C for the composites with a higher content of PU, while the *T_max_* of the composites was higher by 3–5 °C than that of PHB (Table 2).

The thermal stability of the PHB composites was improved due to the addition of PU, which is characterized by a higher thermal stability than PHB. In addition, the elastomeric PU lowered the melt viscosity of the composites by 20–30% compared to that of neat PHB, as indicated by the torque values at the end of melt mixing, and decreased the shear stresses during melt processing and hence the material degradation. A similar increase in the onset degradation temperature by 7 °C was reported for a PHB blend containing 5 wt% poly(3-hydroxyoctanoate) (PHO) and an increase in this temperature by 10 °C for the blend containing 15 wt% PHO [27]. The good interaction between the components, the PHB matrix, the MC filler, and PU could also increase the thermal stability of the composites, as reported in previous works [28,29,30]. Former studies have reported the occurrence of hydrogen bonding interactions between the OH groups of nanocellulose and the carbonyl groups of PHB or PCL, which is a component of the PU elastomer [30,31]. Strong hydrogen bonding interactions may also occur between the NH groups of the isophorone diisocyanate component of PU and the OH/C=O groups in MC and PHB [10,32]. Moreover, the interactions between the components were considered the main cause of the increased thermal stability of PHBV/cellulose nanocrystal composites [30] or PHB composites reinforced with wood waste fibers [29].

Besides the characteristic PHB peaks, an additional shoulder extending over a wide range of temperatures, from 220 °C to 390 °C (Figure 2b), was observed in the DTG diagram of composites. This shoulder shows a maximum close to the *T_maxPU_* located at 350 °C and corresponds to the degradation of PU, which is a slow process. As can be seen in Figure 2b (detail), the peak corresponding to PU degradation (*T_maxPU_* 350 °C) is shifted to higher temperatures in composites, between 370 °C and 390 °C. In addition, no difference was noted between the thermal stabilities of the composites containing 10 wt% PU that were prepared by the two methods (I—masterbatch and II—direct mixing). Therefore, the masterbatch route did not affect the thermal stability of the PHB composites.

### 3.3. Melting and Crystallization Behaviors of the Composites

**Figure 3:**
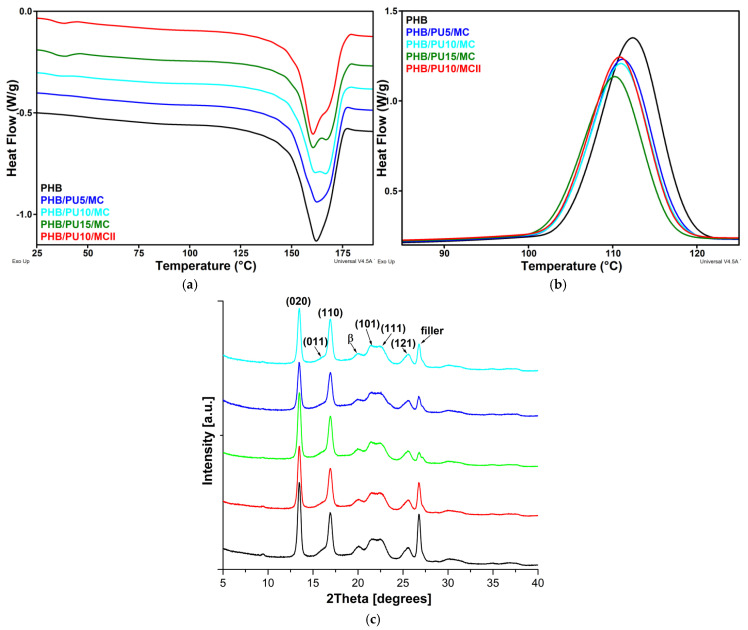
DSC curves from the first heating cycle (**a**) and from cooling (**b**); XRD patterns of PHB and composites (**c**).

The melting (*T_m_*) and crystallization (*T_c_*) temperatures of PHB were slightly influenced by the addition of PU and MC (Figure 3a,b; Table 2). Thus, *T_m_* and *T_c_* of the composites were lower by a maximum of 1.6 °C and 2.2 °C, respectively, than those obtained for neat PHB (Table 2). A similar behavior was reported for PHBV/PU blends [10]. A difference between the thermal behavior of the composites and that of PHB is the occurrence of a shoulder in the melting curves of the composites at temperatures higher than *T_m_* (Figure 3a). This shoulder indicates the bifurcation of the melting peak and appeared more clearly as the PU content in the composites increased. This shows that PU enhanced PHB recrystallization during melting and favored the formation of larger crystals.

A slightly lower crystallization temperature was obtained for composites compared to PHB (Figure 3b). This decrease is determined by the elastomeric nature of the highly flexible PU, which led to a lower crystallization rate along with a lower crystallinity (Table 2). The effect of PU could be considered similar to that of a lubricant or plasticizer, which increases the mobility of the PHB chains up to a lower temperature, hindering its crystallization, as observed for PHB modified with a low-molecular-weight poly(ethyleneglycol) [33] or with acetyl tributyl citrate [34]. However, in contrast to PHB modified with a lubricant or plasticizer, which can increase the mobility of the PHB chains in the amorphous phase, leading to a downshift of its glass transition temperature (*T_gPHB_*), the addition of PU to the composites led to an increase in the *T_gPHB_*. Therefore, PU lowered the crystallization temperature of PHB on the one hand and increased its *T_g_* on the other hand. This is probably due to the low miscibility of PHB with the PCL soft segments of the PU modifier [2]. Indeed, there is a large difference between the melting temperature of the PCL soft segments of PU (36–38 °C) and that of PHB (160–162 °C). This difference is greater than 100 °C and can influence the characteristic temperatures of PHB in blends and composites, similar to previously reported results [35]. It may be assumed that during non-isothermal heating, the PHB/PCL pair, with PCL coming from the soft segments of PU, will change from a crystalline/crystalline system below 36 °C, when both the PHB and PCL are crystalline solids, to a crystalline/amorphous one due to PCL melting above this temperature. Therefore, the presence of PU will decrease the *T_c_* value of PHB in composites because the PCL soft segments have enough mobility at temperatures above the *T_mPCL_* (36 °C), while below this temperature, the crystalline PCL will hinder the motion of the PHB chains, thus increasing the *T_gPHB_*. This behavior is similar to that reported for a PHB/poly(ethylene oxide) (PEO) system, which is a crystalline/amorphous system above the melting point of a PEO blend (60 °C) and a crystalline/crystalline system below 60 °C [35,36]. It should be noted that the highest increase in the *T_gPHB_* of 4.5 °C was noticed for the composite containing 15 wt% PU; therefore, for the highest amount of elastomer used in this work.

Small differences were observed between the thermal behaviors of the two composites containing 10 wt% PU, which were prepared by a masterbatch method and by direct mixing (II). Thus, the degree of crystallinity did not significantly change compared to that of the PHB reference for PHB/PU10/MCII, while X_C_ slightly decreased for PHB/PU10/MC (Table 2). This difference may come from weaker interactions between the components or from worse dispersion of the PU and MC inclusions in the PHB/PU10/MCII.

### 3.4. XRD Analysis of the Composites

The DSC results have shown that PU favored the formation of larger PHB crystals. This effect was verified by XRD measurements. The XRD curves for PHB and its composites (Figure 3c) reveal the characteristic peaks of PHB α-crystals at 2θ 13.5° (020), 16.9° (110), 21.5° (101), 22.6° (111), and 25.5° (121), along with a small peak at about 20°, which corresponds to the PHB β-crystal form, with a zig-zag conformation [37]. The peak at about 26.8° may be ascribed to the diffraction plane (040) of the PHB or to the main diffraction peak of a filler, such as a silicate [38]. The characteristic parameters of the XRD patterns were calculated by fitting curves with Voigt functions using Fityk 1.3.1 software [39]. The crystal size (D) was estimated for the most important peaks corresponding to the diffraction planes (020) and (110) (Table 3) on the basis of the full width at half maximum (β) using the Scherrer equation [40]. A slight increase in crystal size with increasing PU concentration in the composites was observed in directions perpendicular to both considered planes. This observation correlates well with the results obtained by DSC and highlights the influence of PU on the crystallization of PHB in that it leads to the formation of larger crystals and a higher order in the crystalline phase. A similar result was reported for PHB modified with 20 wt% tributyl 2-acetyl citrate or poly(3-hydroxyoctanoate) [34]. In good agreement with the DSC results, a decrease in XRD crystallinity was observed with increasing elastomer concentration in the composites.

The addition of the PU elastomer to PHB may also influence the formation of the β-phase. Although in the case of polypropylene, the percentage of the β-crystal form (K_β_) is, in general, calculated with the Turner–Jones formula [41], we consider that this empirical formula is not suited for PHB, so the amount of PHB β-crystals was calculated by dividing the area of the peak at 2θ 20°, which is ascribed to the β-form, by the sum of the areas of all peaks, corresponding to both α and β crystal forms, and multiplying by 100. The calculated K_β_ values increased from 8.0% for PHB to 9.2% for PHB/PU10/MC and 10.4% for PHB/PU15/MC. Therefore, adding the PU elastomer in increasing quantities to PHB favors the formation of the β-crystal form. This effect may be due to the orientation of the lamellae in the vicinity of the deformed structures of the elastomeric phase. This confinement effect was also observed in polypropylene/ethylene-propylene-diene rubber blends [42].

### 3.5. Dynamic Mechanical Analysis of the Composites

**Figure 4:**
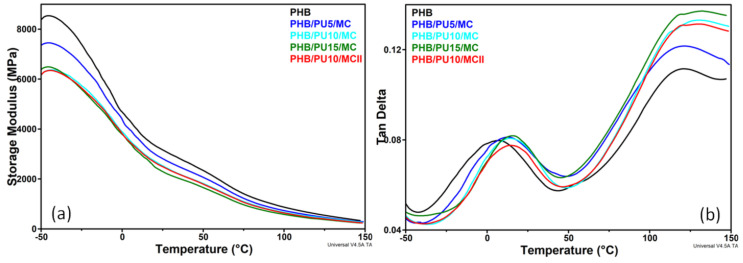
Storage modulus (**a**) and tan delta (**b**) variation with temperature for PHB and composites.

The variation of the storage modulus *(E’*), which is a measure of the energy stored in the sample, with temperature is shown in Figure 4a and the variation of damping (tan δ) is presented in Figure 4b. All the samples underwent a sudden decrease in *E′* value from −40 °C to about 50 °C, which corresponds to the glass transition, and a second slower decline in *E’* value at above 50 °C, which corresponds to the crystal–crystal slip transition [29,43]. While small deviations around 120 °C were noticed for the temperature of the second transition, the glass transition temperature (*T_g_*) determined from the tan δ variation with temperature showed a clear increase for the composites compared to neat PHB and also showed an increase with the PU content in the composites, with a similar trend being obtained by DSC analysis. This increase in the *T_g_* value is determined by the interactions between the components of the composites, PHB, PU, and MC, which leads to a lower mobility of the PHB chains [10,43]. Although both DSC and DMA showed an increase in the *T_g_* value of PHB after PU and MC addition, the temperature jump for the entire series is larger in the DMA measurements, of 8.9 °C vs. 4.5 °C in DSC (Table 4). This is due to the oscillating deformation applied to the sample, which, together with temperature, determines a more significant response of the material to the combined action of the two stimuli. The difference of 20–25 °C between the glass transition temperatures determined through the two methods is due to the difference between the measurement techniques, with DSC measuring the heat flow relative to a reference and DMA measuring mechanical stiffness and energy absorption as a result of the applied oscillation [44,45]. As seen in Table 4, the *E′* values at different temperatures decreased with the increase in the PU concentration in the composites. However, the decrease in stiffness did not exceed 20% at room temperature for the composites obtained by using a masterbatch. A greater decrease in the *E′* value was observed when the PU content increased from 5 to 10 wt% than when it increased from 10 to 15 wt%, which is possibly due to morphological differences between these composites.

Regarding tan δ values, it should be noted that they are much lower than 1 for all samples and temperatures, showing a predominantly elastic behavior [46]. Although the differences between the samples in terms of tan δ peak height are very small, they fall into an increasing trend that correlates well with the increase in the content of the amorphous phase in the composites and the decreased stiffness caused by the addition of the elastomeric PU. In addition, the PHB/PU10/MCII composite presented a lower tan δ peak height, a higher *T_g_* value, and lower storage modulus regardless of the temperature than the counterpart obtained by using the masterbatch (PHB/PU10/MC). These aspects could be related to a higher brittleness [47] and could be better explained if when the morphological peculiarities of the composites are known.

### 3.6. SEM Analysis of Composites

**Figure 5:**
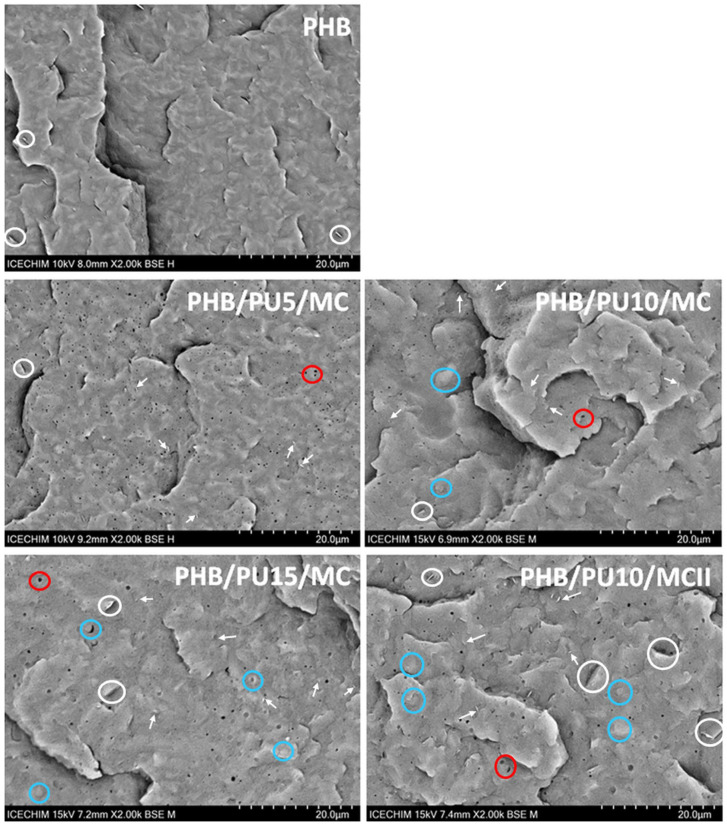
SEM images of cryo-fractured surfaces of PHB and composites.

The SEM analysis (Figure 5) was undertaken to observe the changes in the morphology of the materials, determined by the addition of the PU elastomer and the presence of MC aggregates, if any. The SEM images reveal that a brittle fracture occurred in PHB and composite films after immersion in liquid nitrogen. It should be noted that there were several platelets on the sectioned surface of PHB, which are marked with white circles in Figure 5. They may come from fillers such as nucleating agents that are used in the commercial PHB [48]. No MC aggregates were detected on the fractured surfaces of the composites, only individual nanofibers or assemblies of 2–3 nanofibers, which are marked with white arrows, showing a good dispersion of microfibrillated cellulose in the composites.

The presence of PU elastomers is hardly visible in the SEM images of the composites. PU is not miscible with PHB, with which it forms multiphase systems, consisting of small PU droplets dispersed in the PHB matrix [10]. However, such droplets were not observed in the SEM image of PHB/PU5/MC, and they were hardly visible in the composites with 10 and 15 wt% PU, being marked with blue circles in Figure 5. It can be assumed that the presence of MC ensured better dispersion of the elastomeric phase in the composites, which made the droplets no longer visible, as previously observed for other PHBV/PU systems [12]. However, larger and poorly distributed formations, which can be attributed to the PU modifier, were observed in the SEM image of PHB/PU10/MCII. The direct mixing of the components instead of using a masterbatch could be the cause of this observation.

The presence of small voids of submicron and nanometer size was observed in all the composites and marked with red circles (Figure 5). Their origin is difficult to determine as they may come from water or volatiles that left the system at the high temperatures used during processing or from pull-out elastomeric particles or MC fibers. Regardless of their origin, the SEM images show that the voids are evenly distributed in the composites section and that their average size is higher for PHB/PU15/MC and PHB/PU10/MCII (0.42 µm ± 0.12 and 0.35 µm ± 0.11) than for PHB/PU5/MC and PHB/PU10/MC (0.22 ± 0.10 µm and 0.29 ± 0.11 µm).

### 3.7. Hydrolytic Degradation of the Composites

#### 3.7.1. Mass Loss During Hydrolytic Degradation

The chemical hydrolysis of PHB was studied, especially for biomedical applications, such as bioresorbable implants [49,50], and for chemical recycling [51]. It can be assumed that the addition of microfibrillated cellulose and polyurethane in PHB will influence the hydrolytic degradation of composites. However, the hydrolysis of PHB in physiological conditions at pH = 7.4 is a very slow process, while its hydrolysis in an alkaline environment is much faster [20,52,53]. In addition, enzymatic degradation of PHB by several degrading enzymes occurs at a slightly alkaline pH [52,54]. Therefore, in this work, the hydrolytic degradation of PHB/PU/MC composite films was, for the first time, studied and compared to that of neat PHB in an alkaline environment at pH = 13 and at 25 °C and 50 °C for 28 days.

**Figure 6:**
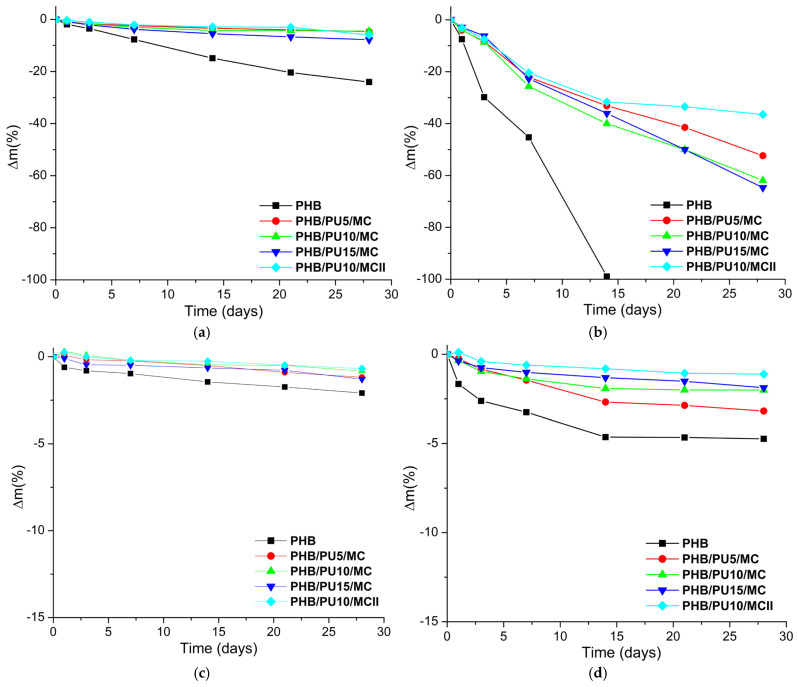
Mass loss percentage during hydrolytic degradation of neat PHB and composite films in an alkaline environment at 25 °C (**a**) and 50 °C (**b**) and in an acidic environment at 25 °C (**c**) and 50 °C (**d**).

The mass loss during hydrolytic degradation is presented in Figure 6. The degradation of neat PHB in an alkaline environment at 25 °C was much higher than that of the composites, with a mass loss of 24% being obtained for PHB while it was under 8% for the composites. Several mechanisms have been elaborated so far for the hydrolytic degradation of PHB, with one of them considering PHA hydrolysis as a surface erosion process [55], others considering the degradation as taking place mainly in the bulk of the PHB samples [20,22], and another considering the hydrolytic degradation of PHB as a two-step process, with the bulk of the degradation taking place after an induction period that allows water to penetrate inside the sample [56].

The differences between the reported data may occur because the hydrolytic degradation of PHB depends on many factors, including hydrolysis conditions (pH, temperature, pressure, and time), polymer properties (crystallinity, molecular weight, porosity, and pore size), the form and characteristics of the sample (foil, granules, bars, thickness, size, and others), and processing conditions (compression molding, injection molding, and solution casting) [52,57]. In one case, the surface erosion mechanism for the abiotic hydrolytic degradation of PHBV was established based on the non-uniform variation of water uptake with root time and sample thickness [55], but the test was conducted under conditions that may not illustrate the real situation for the hydrolytic degradation of PHBV. Therefore, the abiotic degradation of PHB may be largely considered as a bulk process.

The hydrolytic degradation of PHB occurs at the ester bonds, similar to other polyesters [55,57]. Following its hydrolytic degradation, only monomers in high proportions and dimers in low proportions were found after extraction, showing that the hydrolytic degradation occurs mainly from the end of the PHB chains [20]. In addition, the mass loss is influenced by the diffusion rate of the degradation products, with the diffusion of the monomer being faster than that of water-soluble oligomers, if they have not already been hydrolyzed by the alkaline environment [20,56]. The addition of a polyurethane, which is generally characterized by a very slow degradation in different environments [14], will decrease the rate of hydrolytic degradation of the composites, as observed in Figure 6a. Although the PU used in this study contains a biodegradable poly(ε-caprolactone) soft segment, which undergoes hydrolytic degradation under certain conditions [58,59], the high hydrophobicity of PCL segments prevents water penetration and delays degradation [60]. As seen in Figure 6a, the concentration of PU did not lead to significant differences regarding the mass loss of the composites PHB/5PU/MC and PHB/10PU/MC, while the composite with 15 wt% PU showed a slightly greater mass loss than the other composites. The slightly higher mass reduction during the degradation of PHB/15PU/MC in an alkaline medium is probably due to the different porosity of this composite compared to the others, especially to its larger pore size, as determined from the SEM images. A higher porosity or larger pores may favor hydrolytic degradation due to the easier penetration and diffusion of water through the material [61,62].

The hydrolytic degradation for PHB and composite films in alkaline media at 50 °C (Figure 6b) took place at a higher rate, resulting in a much higher mass loss for all samples than in the same test at 25 °C. Thus, after 28 days, the PHB film was almost completely degraded, while the composites obtained by the masterbatch method showed mass losses between 53 and 65% (53%, 62%, and 65% for the composites with 5 wt%, 10 wt%, and 15 wt% PU). The almost complete degradation of PHB under strong alkaline conditions at 50 °C could be due to several factors, including the formation of sodium 3-hydroxybutyrate, which is soluble in water [63,64], and the increased mobility of molecular chains at a higher temperature, which favors chain scission reactions [65]. Indeed, the increase in temperature accelerated the hydrolytic degradation of all samples, both PHB and composites. Previously reported data have shown that the increase in temperature strongly enhanced the degradation rate in different media and conditions [22,55,56,66]. A more than three-fold increase in the mass loss of poly(4-hydroxybutyrate) (P4HB) and a four-fold decrease in its molecular weight were obtained when P4HB films were subjected to hydrolytic degradation at 55 °C compared with 37 °C in an alkaline medium with a pH of 10 for 14 days [22]. A significant influence of temperature on the hydrolytic degradation of PHB was also reported for PHB films immersed in an alkaline medium with a pH of 14 for 100 h: 35% mass loss was obtained at 30 °C, 60% at 35 °C, and 90% at 40 °C [66]. Moreover, abiotic depolymerization of PHBV in conditions similar to those used in this study (pH 13, 60 °C) led to 98.1% soluble degradation products (based on the original mass of PHB), which were recovered after hydrolysis [67].

The addition of PU and MC in PHB composites led to the delay of hydrolytic degradation at 50 °C (Figure 6b). This was determined by the different behavior of PU and MC as compared to PHB in an alkaline environment at this temperature. Both PU and MC can be hydrolyzed to some extent in alkaline conditions, but at a slower rate [68,69,70,71,72]. The hydrolytic degradation of thermoplastic polyurethanes depends on a great number of factors including the type and length of hard and soft domains, crystallinity, the ratio between soft and hard segments, and the pH of the environment, to name only a few [68,69,70]. A segmented polyurethane prepared from polycaprolactone diol, ethylene glycol, dimethylolpropionic acid, and hexamethylene diisocyanate, with a 30% hard segment fraction, lost less than 10% of its mass following hydrolytic degradation for 8 weeks in phosphate buffer saline at 37 °C [69], while a poly(ε-caprolactone-*co*-β-butyrolactone) (PCLBL)-based polyurethane with a 90.5/9.5 CL/BL molar ratio lost between 40 and 50% of its mass after hydrolytic degradation for 2 weeks in NaOH aqueous solution (pH~12) at 45 °C [70]. Although this latter mass loss value is relatively close to our results, the molecular weight of the PCLBL-based polyurethane was four times lower than that of the PU used in our work, which certainly favored hydrolytic degradation. The complete degradation of some polyurethanes was achieved in much harsher conditions, namely 0.2 g/mL NaOH and 80 °C, and was proposed as a solution for the chemical recycling of waste polyurethane [73]. Regarding the hydrolytic degradation of cellulose in an alkaline environment, it is generally accepted that it is only partial even at 0 °C [71,72]. Therefore, it can be assumed that the PU and MC will be less affected by the hydrolytic degradation and the undegraded PU and MC residues will reduce the mass loss, leading to lower values in the case of composites, as can be seen in Figure 6b.

A lower mass loss was observed after 28 days of alkaline hydrolytic degradation for PHB/10PU/MCII compared to PHB/10PU/MC (Figure 6b). This behavior may be explained by the poorer dispersion and larger size of the PU inclusions in PHB/10PU/MCII, as determined by the direct mixing route adopted in this case and also by the higher PHB crystallinity of PHB/10PU/MCII compared to PHB/10PU/MC, which reduced the hydrolytic degradation ability of PHB [52,66].

For comparison, hydrolysis of PHB and composite films was also conducted in an acidic environment (pH = 1) at 25 °C and 50 °C for 28 days, and the mass loss is shown in Figure 6c,d. Much lower mass losses were obtained in the HCl environment compared to the alkaline one, i.e., below 2.5% at 25 °C and below 5% at 50 °C. Previous works have reported similar observations [22,74,75,76,77]. Analyzing the formation of the hydrolytic products 3-hydroxybutyric acid and crotonic acid, Yu et al. [74] reported that PHB endured hydrolysis under moderate acid conditions but was strongly attacked in an alkaline environment. Similarly, a poly(3-hydroxybutyrate)/poly(ethylene glycol) graft copolymer (PHB/5%PEG) showed a mass loss close to 6% after 30 days of treatment in an acidic environment (pH = 1) at 37 °C but displayed a much higher mass loss (~45%) when exposed to an alkaline treatment (pH = 13) [75]. This behavior has been explained by the ability of hydroxyl anions to decrease the energy barrier of ester bond splitting and the low probability of re-esterification [74].

#### 3.7.2. Thermal Stability of Degraded Samples

**Figure 7:**
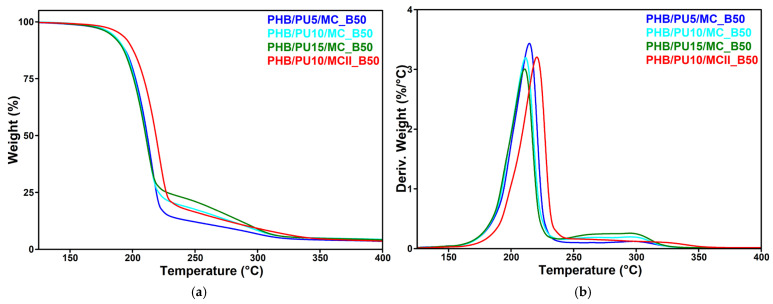
TGA (**a**) and DTG (**b**) curves of PHB/PU/MC composites after hydrolytic degradation in an alkaline environment at 50 °C for 28 days.

The TGA and DTG diagrams of the composites after hydrolytic degradation in an alkaline environment at 50 °C for 28 days are shown in Figure 7a,b. Some interesting aspects were observed when the characteristic data extracted from the TGA-DTG diagrams after alkaline hydrolysis (Table 5) were compared with those before exposure (Table 2).

As can be seen in Table 2 and Table 5, the *T_10%_* and *T_max_* of the nanocomposites strongly decreased after alkaline hydrolysis at pH 13: *T_10%_* decreased by 58–65 °C and *T_max_* decreased by 67–76 °C. The lower thermal stability of nanocomposites after alkaline hydrolysis at 50 °C is due to the hydrolytic cleavage of the ester group in PHB under the nucleophilic attack of hydroxide ions [20,77]. This is a well-documented process that occurs mostly at the ends of the chains, leading to the generation of the monomer and, to a lesser extent, other oligomers [20,74].

It can be remarked that the PU concentration had only a small influence on the thermal degradation of hydrolyzed nanocomposites, while the processing route had a greater influence, with the sample obtained by direct mixing being characterized by higher thermal stability than the sample obtained by the masterbatch route. This behavior may be related to the higher degree of crystallinity of PHB/PU10/MCII compared to PHB/PU10/MC (see Table 2). Indeed, previous studies have shown that a lower crystallinity may contribute to the accelerated biodegradation of PHB [66,75,78]. Tapadiya and Vasanthan [66] reported that PHB films exposed to annealing at different temperatures resulted in PHB samples with different crystallinity, and the degradation of annealed films by alkaline hydrolysis decreased with increasing their degree of crystallinity. A similar observation was reported by Zhijiang et al. [75] for a poly(3-hydroxybutyrate)/poly(ethylene glycol) graft copolymer, which showed much faster degradation with increasing degrees of grafting and, correspondingly, with decreasing crystallinity.

#### 3.7.3. Morphological Analysis of the Degraded Samples

**Figure 8:**
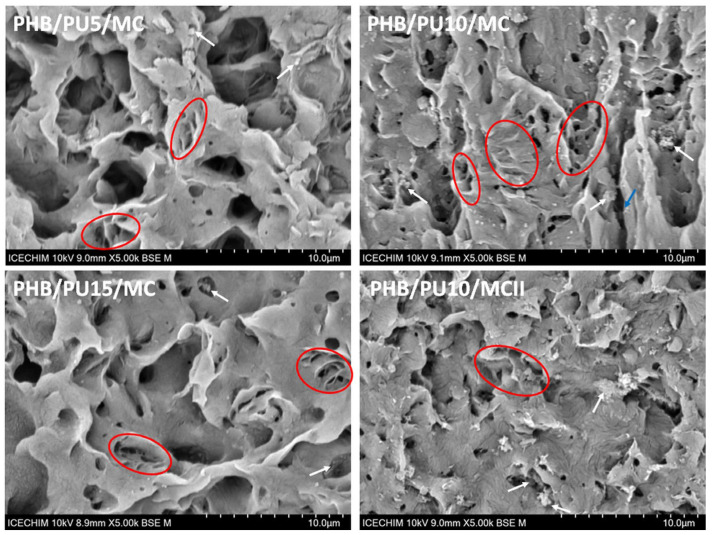
SEM images showing the surface morphology of the composites after exposure to hydrolytic degradation for 28 days at 50 °C.

The representative SEM images in Figure 8 show the surface morphology of composites after hydrolytic degradation at pH 13 and 50 °C for 28 days. Clear signs of degradation were observed in all composites regardless of composition or procedure, but the intensity of degradation varied from sample to sample. PHB/PU5/MC showed the largest and deepest cavities in the bulk of the material, which indicates the most advanced hydrolytic degradation among composites. This composite contains the largest amount of PHB (93 wt%); therefore, it can be assumed that the PHB was the most-attacked component from the composite. As can be seen in Figure 8, the alkaline water selectively attacked the material and penetrated the composite, digging deep cavities and cracks (marked with blue in Figure 8) and substantially modifying the internal morphology, as also observed for other PHB blends [75]. Previous works have reported that the ester bonds, especially those at the end of the PHB chains, are the most susceptible to hydrolyzation, leading to macromolecular chain fragmentation and releasing soluble monomers and small oligomers [20,22,74].

Although both amorphous and crystalline domains are degraded following hydrolysis [56,66], previous studies have shown that the degradation of the amorphous phase is faster than that of the crystalline phase [66,74,77]. Therefore, it is possible to see the crystalline arrangements after alkaline hydrolysis. In a recent work [22], enzymatic degradation with selected enzymes was used to disclose the spherulitic morphology of P4HB after surface erosion of the amorphous domains. The PHB/PU/MC composites, which contain about 60% amorphous PHB (Table 2), have a consistent fraction of amorphous regions that are more prone to degradation than the crystalline ones. Indeed, lamellar bundles (circled with a red line in Figure 8), which remained after hydrolytic degradation, were observed in the SEM images of the composites. They are part of the ridge–valley crystalline structures of PHB [35,79,80], which can be better discerned on the PHB/10PU/MC surface, where several vertical ridge–valley arrangements can be observed (Figure 8—PHB/10PU/MC). The fragments marked with white arrows in Figure 8 may be ascribed to the PU and MC modifiers, which were not affected to the same extent by the attack of the alkaline solution.

In general, the remnants of the crystalline order (lamellar bundles and ridge–valley arrangements) were frequently seen in the SEM images of PHB/PU5/MC, PHB/PU10/MC, and PHB/PU15/MC, and hardly observed in the case of PHB/PU10/MCII (Figure 8). This is probably due to PHB/PU10/MCII having the highest crystallinity among the composites. A higher crystallinity prevents the penetration of water and the removal of soluble hydrolysis products. In addition, the presence of agglomerations or the uneven dispersion of PU, which is known to repel water [81,82], also prevents the degradation of the amorphous PHB. These observations correlate well with the lower mass loss obtained for PHB/PU10/MCII compared to the other composites (Figure 6). The influence of the PU modifier on the hydrolytic degradation of PHB composites may also be observed from the aspect of the holes in the samples with increasing PU content. Thus, smaller and, in general, shallower holes were observed in the SEM images of the composites containing larger amounts of PU (Figure 8). Indeed, the analysis of the approximate size of the holes using ImageJ software version 1.8.0 showed an average hole size of 2.85 ± 1.51 µm for PHB/PU5/MC, 1.55 ± 1.15 µm for PHB/PU10/MC, and 1.38 ± 0.87 µm for PHB/PU15/MC.

**Figure 9:**
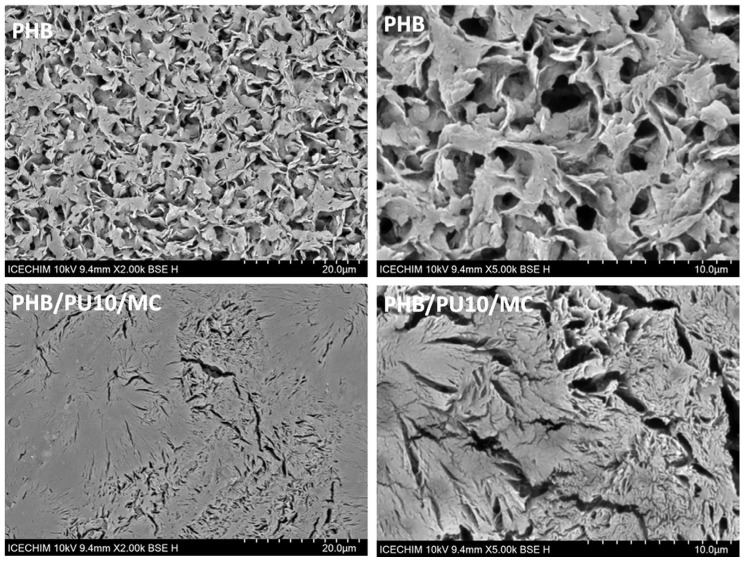
SEM images with different magnifications (×2000—left and ×5000—right) for PHB and the PHB/10PU/MC composite after exposure to hydrolytic degradation at 25 °C for 28 days.

The hydrolytic degradation of PHB at 50 °C for 28 days led to its almost complete degradation, but the PHB film kept its integrity when the alkaline hydrolysis was conducted at 25 °C for the same period. For a more robust comparison, SEM images with different magnifications of the surface of PHB and PHB/PU10/MC films previously exposed to alkaline hydrolysis at 25 °C for 28 days are shown in Figure 9.

Important differences between the degradation rates of PHB and composites can be observed in these images. The surface of PHB was already dug by the alkaline water, probably through the attack on the amorphous areas, leaving the crystalline ones less affected, which leads to a specific floral appearance (Figure 9).

Contrarily, the PHB/10PU/MC surface showed only cracks that seem to be distributed according to the shape of the spherulites. This aspect clearly shows that the amorphous areas surrounding the crystalline spherulites were first attacked by the alkaline solution. The slower attack on the composite surface compared to PHB may be due to the presence of PU, which prevents the penetration of water and protects the surface of the composite, as discussed above. The influence of microfibrillated cellulose should not be excluded since it creates a network and tortuosity that also prevents water penetration in the bulk of the composite [83]. Delayed degradation under compost conditions was also obtained for a PLA reinforced with microcrystalline cellulose, with the neat PLA needing only 4 weeks to begin degrading while the composite took more than 8 weeks [84].

The results of accelerated degradation in alkaline conditions have shown that the PU modifier, and possibly the MC, delayed PHB degradation by 12 days in the case of PHB/15PU/MC, and by even longer for the other samples, if the degradation rate is maintained after the period of 28 days. However, degradation in natural environments should also be undertaken because these composites are promising substitutes for synthetic polymers derived from petroleum resources for both medical and engineering applications.

## 4. Conclusions

In this work, PHB was modified with microfibrillated cellulose and a thermoplastic polyurethane containing biodegradable segments to overcome some of its drawbacks. The composites were obtained by two routes, using a masterbatch and by direct mixing. The PU, which was added in different concentrations in the composites, and MC modifiers improved the thermal stability of PHB by up to 13 °C and slightly decreased its melt viscosity and crystallinity, thus improving the melt processability of the composites. The addition of a PU/MC masterbatch to PHB led to a decrease in the storage modulus, which did not exceed 20% at room temperature. An increase in the glass transition temperature by up to 9 °C was obtained with increasing PU content in the composites, showing possible interactions between the components, PHB, PU, and MC. Hydrolytic degradation of the composites in an alkaline environment at 50 °C for 28 days determined a strong decrease in the thermal stability by 58–65 °C compared to the undegraded composites. The mass loss and morphological investigation showed that the PU modifier delayed the degradation of PHB composites by more than 12 days if the degradation rate was maintained after the period of 28 days. The improved thermal stability, melt processability, and lower cost, along with higher flexibility and the possibility of controlling hydrolytic degradation by the PU content, make the PHB/PU/MC composites obtained by the masterbatch method promising materials for medical and engineering applications.

## Figures and Tables

**Table 1:** Composition of the PHB/PU/MC composites expressed in wt%.

Composites	PHB	Masterbatch (PU/MC = 5/2)	PU	MC
PHB	100	-	-	-
PHB/PU5/MC	93	7	-	-
PHB/PU10/MC	88	7	5	-
PHB/PU15/MC	83	7	10	-
PHB/PU10/MCII *	88	-	10	2

* Method II, without masterbatch.

**Table 2:** TGA and DSC results: temperature at 10% weight loss (*T_10%_*), temperature at the maximum degradation rate (*T_max_*), residue at 700 °C (*R_700_*), glass transition of the PHB (*T_gPHB_*) and PCL from PU (*T_gPCL_*), melting (*T_m_*) and crystallization (*T_c_*) temperatures of PHB, and the degree of crystallinity (*X_C_*).

Composites	*T_10%_* °C	*T_max_* °C	*R_700_* %	*T_gPHB_* °C	*T_mPCL_* °C	*T_m_* °C	*T_c_* °C	*X_C_* %
PHB	244.1	282.5	0.9	−13.4	-	162.1	112.4	41.2
PHB/PU5/MC	251.0	287.7	1.1	−13.2	-	162.4	111.1	40.2
PHB/PU10/MC	256.6	287.7	1.0	−12.6	35.7	161.7	111.0	39.1
PHB/PU15/MC	255.6	285.8	1.0	−8.9	38.1	160.6	110.2	39.3
PHB/PU10/MCII	255.8	287.1	1.0	−11.1	37.9	160.5	110.9	41.5

**Table 3:** Full width at half maximum of the peak (β), and crystal size (D) in the direction perpendicular to (020) and (110) planes along with the XRD crystallinity (X_XRD_) of composites.

Composites	Miller Indices	β (Radian)	D (nm)	X_XRD_ (%)
PHB	(020) (110)	0.00680 0.00850	20.5 16.5	71.0
PHB/PU5/MC	(020) (110)	0.00668 0.00830	20.9 16.9	66.5
PHB/PU10/MC	(020) (110)	0.00660 0.00824	21.2 17.0	62.6
PHB/PU15/MC	(020) (110)	0.00660 0.00811	21.2 17.3	56.7
PHB/PU10/MCII	(020) (110)	0.00660 0.00823	21.2 17.0	63.1

**Table 4:** Dynamic mechanical properties of nanocomposites: *T_g_*, storage modulus (E’) at −25, 25, and 100 °C, and tan δ peak height (tan δ).

Samples	*T_g_,* °C	tan δ	*E′*_−25°C_*,* MPa	*E′*_25°C_, MPa	*E′*_100°C_, MPa
PHB	6.3	0.080	7388	3165	871
PHB/PU5/MC	10.6	0.081	6577	2816	734
PHB/PU10/MC	12.8	0.081	5735	2528	662
PHB/PU15/MC	15.2	0.082	5660	2512	659
PHB/PU10/MCII	14.5	0.078	5592	2296	582

**Table 5:** TGA and DTG results for the nanocomposites exposed to hydrolytic degradation for 28 days in an alkaline environment at 50 °C; *T_10%_*, *T_max_*, and *R_700_* have the same meaning as in Table 2.

Composites	*T_10%_* °C	*T_max_* °C	*R_700_* %
PHB/PU5/MC	191.3	214.7	1.37
PHB/PU10/MC	191.4	211.6	1.51
PHB/PU15/MC	190.4	210.8	1.60
PHB/PU10/MCII	198.1	220.6	1.21

## Data Availability

Data are contained within the article.
